# *Panax ginseng* Leaf Extracts Exert Anti-Obesity Effects in High-Fat Diet-Induced Obese Rats

**DOI:** 10.3390/nu9090999

**Published:** 2017-09-10

**Authors:** Seul Gi Lee, Yoon Jeong Lee, Myeong-Hwan Jang, Tae Ryong Kwon, Ju-Ock Nam

**Affiliations:** 1 Department of Food Science and Biotechnology, Kyungpook National University, Daegu 41566, Korea; lsg100479@naver.com; 2 Department of Life Sciences, Yeungnam University, Gyeongsan 38541, Korea; yjlee-7447@hanmail.net; 3 Punggi Ginseng Research Institute Gyeong Buk A.R.E.S, Gyeongsangbuk-do Agricultural Research & Extension Services, Daegu 41404, Korea; hwanmj@korea.kr (M.-H.J.); trkwon1177@korea.kr (T.R.K.); 4 Division of Agriculture Environment Research, Gyeongsangbuk-do Agricultural Research & Extension Services, Daegu 41404, Korea; 5 Institute of Agricultural Science & Technology, Kyungpook National University, Daegu 41566, Korea

**Keywords:** 3T3-L1 adipocytes, anti-adipogenic, anti-obesity, leaf extracts, *Panax ginseng*

## Abstract

Recent studies have reported that the aerial parts of ginseng contain various saponins, which have anti-oxidative, anti-inflammatory, and anti-obesity properties similar to those of ginseng root. However, the leaf extracts of Korean ginseng have not yet been investigated. In this study, we demonstrate the anti-obesity effects of green leaf and dried leaf extracts (GL and DL, respectively) of ginseng in high-fat diet (HFD)-induced obese rats. The administration of GL and DL to HFD-induced obese rats significantly decreased body weight (by 96.5% and 96.7%, respectively), and epididymal and abdominal adipose tissue mass. Furthermore, DL inhibited the adipogenesis of 3T3-L1 adipocytes through regulation of the expression of key adipogenic regulators, such as peroxisome proliferator-activated receptor (PPAR)-γ and CCAAT/enhancer-binding protein (C/EBP)-α. In contrast, GL had little effect on the adipogenesis of 3T3-L1 adipocytes but greatly increased the protein expression of PPARγ compared with that in untreated cells. These results were not consistent with an anti-obesity effect in the animal model, which suggested that the anti-obesity effect of GL in vivo resulted from specific factors released by other organs, or from increased energy expenditure. To our knowledge, these findings are the first evidence for the anti-obesity effects of the leaf extracts of Korean ginseng in vivo.

## 1. Introduction

Obesity is a major public healthy problem and is associated with an increased incidence of metabolic diseases worldwide [1,2]. It is caused by an imbalance between energy intake and energy expenditure, which generates excessive adipose tissue [3,4]. Adipose tissue mass can be regulated by the adipogenesis process or adipocyte differentiation, which has been extensively studied for the treatment or prevention of obesity [5,6]. Although many studies have investigated potential treatments for obesity, the development of drugs for long-term obesity treatment has not been very successful [7]. Therefore, strategies for the prevention of obesity using health functional foods are considered important.

The root of ginseng has been traditionally used for the prevention and treatment of various diseases for over 2000 years. Root extracts of Korean ginseng (*Panax ginseng* C.A. Meyer) have been reported to exhibit anti-adipogenic activity in 3T3-L1 adipocytes; these extracts were found to contain quantifiable amounts of ginsenosides such as ginsenoside Rg1, Re, Rf, Rb1, and Rc [8]. However, the aerial parts of ginseng have not been effectively utilized. Previous studies have reported that the leaf of ginseng is rich in bioactive compounds and that the content of some ginsenosides in it is higher than that found in the roots [9]. In addition, extracts of the stem, leaf, and berry of American ginseng (*Panax quinquefolium*) have been shown to exhibit anti-obesity activities in vivo [10,11,12].

A previous study has demonstrated that mature (aged) leaves of American ginseng have a higher content of total ginsenosides than that in young leaves [13]. One-month-old leaves contained a total ginsenoside content of 1.33–2.64 g/100 g, while four-month-old leaves contained a total ginsenoside content of 4.14–5.58 g/100 g.

However, there is a lack of reports on pharmacological effects of the aerial parts of Korean ginseng in comparison to American ginseng and anti-obesity effects of leaf extracts of Korean ginseng have not yet been investigated.

Therefore, we examined the anti-obesity effects of leaf extracts from Korean ginseng in high-fat diet (HFD)-induced obese rats. Additionally, the effects of old and young leaf extracts were compared. The results showed that the leaf extracts of Korean ginseng have anti-obesity effects in vivo and in vitro; however, the mechanisms of action were presumed to be different for the extracts.

## 2. Materials and Methods

### 2.1. Preparation of Ginseng Leaf Extracts

Leaf extracts of ginseng (three years old) were provided by the Gyeongsangbuk-do Agricultural Research and Extension Services (Korea). In this study, the extracts of young and old leaves are referred to as extract of green leaf (GL) and dried leaf (DL), respectively. Two different types of leaves were collected and dried at 50 °C for 24 h. Then, the leaf samples were extracted with distilled water (a dried sample/water ratio of 10:3 (*w*/*w*)) at 90 °C for 72 h and stored at 4 °C for further use.

### 2.2. Animals

Five-week-old male Sprague-Dawley (SD) rats were purchased from Hyochang Science (Korea). After a 1-week adaptation period, the animals were randomly assigned to the following groups, each consisting of seven animals: (1) normal diet (ND); (2) HFD; (3) HFD + GL supplementation (3.3 mg/kg); and (4) HFD + DL supplementation (3.3 mg/kg). The composition of the HFD is shown in Table S1, and all rats were maintained under a 12 h light-dark cycle. GL and DL were orally administered on each day of the experimental period. After the treatment was completed, samples of blood and adipose tissues were collected for further analysis. All protocols involving animal experiments were approved by the Institutional Animal Care Committee of Kyungpook National University (approval number: KNU 2017-13 and approval date: 2 February 2017).

### 2.3. Biochemical Analysis

On the final day of the experiments, blood was collected into serum collection tubes and centrifuged at 3000 rpm for 15 min to obtain the plasma supernatant. The plasma marker levels for nephrotoxicity, hepatotoxicity test, and lipid profiling were determined using an automatic analyzer.

### 2.4. Cell Culture and Differentiation

The 3T3-L1 mouse preadipocyte cells were purchased from the Korea Cell Line Bank (KCLB, Seoul, Korea) and cultured in Dulbecco’s modified Eagle medium (DMEM; GIBCO, Grand Island, NY, USA) supplemented with 10% bovine calf serum (BCS; GIBCO). To induce differentiation, the 3T3-L1 adipocytes were cultured to post-confluence (designated as Day 0) for 2 days and the media were replaced with DMEM containing 10% fetal bovine serum (FBS; GIBCO) and MDI solution (composition: 0.5 mM IBMX, 1 μM DEXA, 0.125 mM indomethacin, and 10 μg/mL insulin) for 2 days. Thereafter, the cells were maintained in media supplemented with 10% FBS and 10 μg/mL insulin for 4 days. To examine the anti-adipogenic effects of the extracts, the cells undergoing differentiation were treated with GL and DL during all stages of differentiation.

### 2.5. Oil Red O Staining and Triglyceride (TG) Assay

Oil Red O staining (ORO) and TG assay were performed as previously described [14]. Briefly, cells that had undergone differentiation into mature adipocytes were washed, fixed, and stained with Oil Red O solution to detect lipid droplets. All images were obtained by using a microscope (Leica, Wetzlar, Germany). The TG assay was performed in accordance with the manufacturer’s instructions.

### 2.6. Cell Viability (MTT Assay)

The 3T3-L1 preadipocytes were seeded into 96-well plates and treated with GL or DL at a concentration of 0.15 or 0.3 mg/mL for 48 h. To examine the effect of GL or DL on mature adipocytes, 3T3-L1 cells were seeded and treated with MDI and with GL or DL for the entire differentiation period. At the end of the treatment, the medium was removed and replaced with MTT (3-(4, 5-dimethylthiazol-2-yl)-2, 5-diphenyltetrazolium) solution and incubated for 3 h at 37 °C. After incubation, the precipitated formazan was dissolved with isopropyl alcohol (Duksan Pure Chemicals, Seoul, Korea).

### 2.7. Reverse Transcription-Polymerase Chain Reaction (RT-PCR)

RNA was isolated using RNAiso Plus reagent (TaKaRa Bio, Shiga, Japan). Complementary DNA was synthesized using the Prime Script RT reagent kit (TaKaRa Bio) in accordance with the manufacturer’s protocol. Specific rat and mouse primers were designed, which are shown in Table S2. mRNA expression in 3T3-L1 adipocytes and epididymal adipose tissues was normalized to that of β-actin and GAPDH, respectively, by using ImageJ software (National Institutes of Health, Bethesda, MD, USA).

### 2.8. Western Blot

Total protein was extracted using PRO-PREP lysis buffer (iNtRON Biotechnology, Seongnam, Korea), which contains phosphatase inhibitors and a protease inhibitor cocktail. The lysates were centrifuged at 13,000 rpm for 15 min. The protein samples were separated by SDS-PAGE, transferred onto nitrocellulose membranes, blocked using 5% non-fat skim milk, and incubated overnight at 4 °C with antibodies against PPARγ, LPL, aP2, adiponectin (Abcam, Cambridge, UK), C/EBPα (Cell Signaling Technology Beverly, MA, USA), and β-actin (Santa Cruz Biotechnology, Santa Cruz, CA, USA).

### 2.9. Statistical Analysis

Data are expressed as mean ± SD. Statistical comparisons were performed using one-way ANOVA. Values of *p* < 0.05 were considered statistically significant (* *p* < 0.05 and ** *p* < 0.01).

## 3. Results

### 3.1. Effects of GL and DL on Body Weight, Adipose Tissue Mass, and Food Intake in HFD-Induced Obese Rats

**Figure 1:**
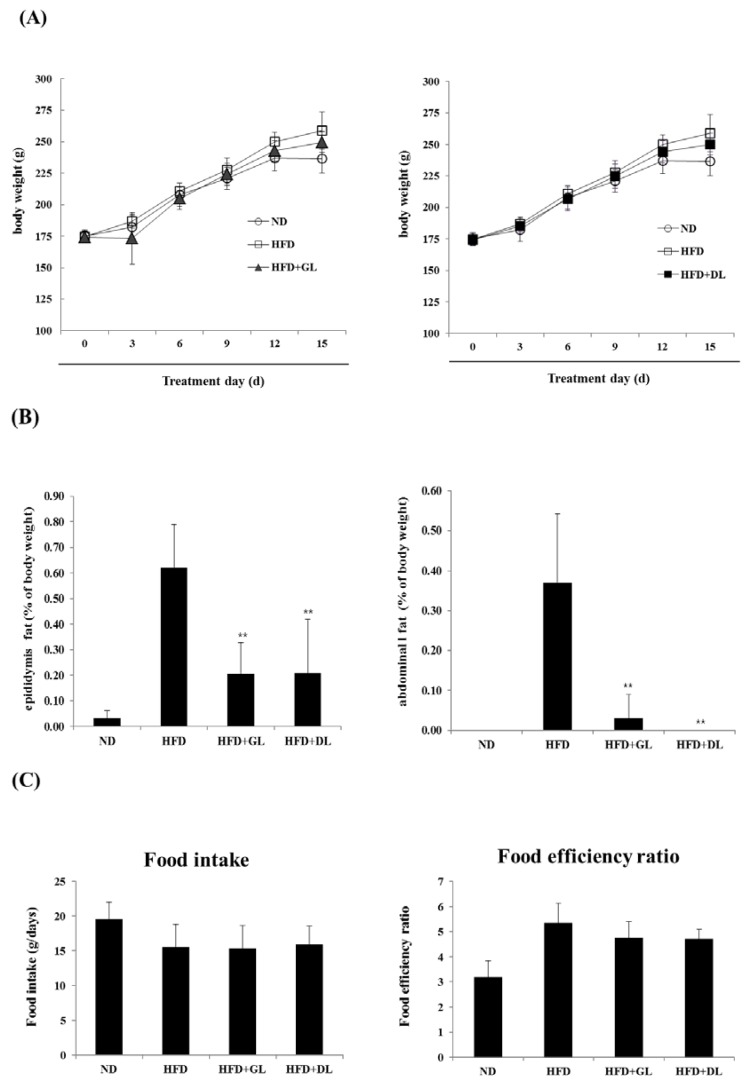
Effects of GL and DL on body weight, adipose tissue mass, and food intake in high-fat diet (HFD)—induced obese rats. The rats (*n* = 7 per group) were fed an ND or HFD. Additionally, HFD-fed rats were also treated with GL or DL at a dose of 3.3 mg/kg/day. (**A**) Body weight was measured every three days; (**B**) total epididymal WAT and abdominal WAT weight; and (**C**) food intake and food efficiency ratio. Significantly different from the HFD group, ** *p* < 0.01 and * *p* < 0.05. Bar graphs show the mean ± SD from seven individual rats per group.

To examine the biological effectiveness of GL and DL, we confirmed the anti-obesity effects of GL and DL in diet-induced obese rats. The final body weights of GL- and DL-supplemented rats (HFD-GL and HFD-DL rats) were slightly lower than those of HFD rats that did not receive supplementation (HFD-fed rats); however, these differences were not significant (Figure 1A). The mass of abdominal and epididymal adipose tissues significantly decreased in HFD-GL and HFD-DL rats compared with that in HFD-fed rats (Figure 1B). Additionally, HFD-GL and HFD-DL rats showed a decreased food efficiency ratio, but the differences were not significant (Figure 1C).

### 3.2. Effects of GL and DL on Activity Levels of Serum Marker Enzymes

**Figure 2:**
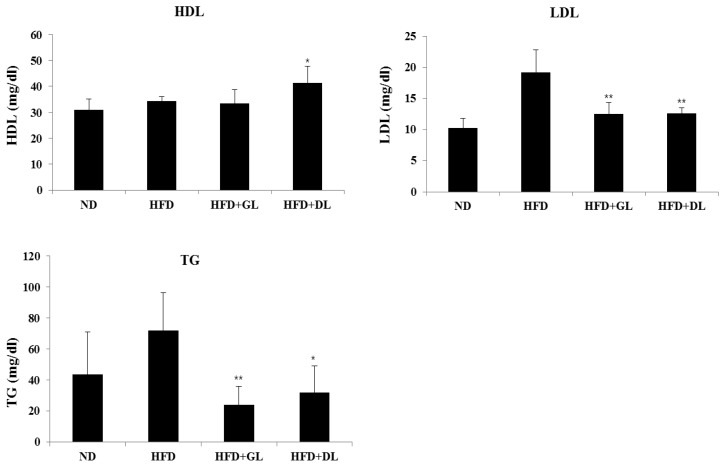
Plasma biochemical values in male rats. Blood plasma were collected and analyzed for TG (triglycerides), HDL (high-density lipoprotein cholesterol), and LDL (low-density lipoprotein cholesterol). Significant differences from the positive control group (HFD), ** *p* < 0.01 or * *p* < 0.05, are indicated. Bar graphs show the mean ± SD from seven individual rats per group.

To evaluate the safety of GL and DL, we confirmed the nephrotoxic and hepatotoxic effects of GL and DL. The levels of ALB, TBIL, BUN, and CRE were not significantly different between HFD-fed rats and HFD-GL or HFD-DL rats (Table S3). As expected, GL and DL did not affect the weights of other organs compared with those in the HFD rats (Table S4). Furthermore, GL and DL increased HDL-cholesterol and reduced LDL-cholesterol and TG levels (Figure 2).

### 3.3. Effects of GL and DL on the mRNA and Protein Expression of Adipogenic Genes in Adipose Tissue

**Figure 3:**
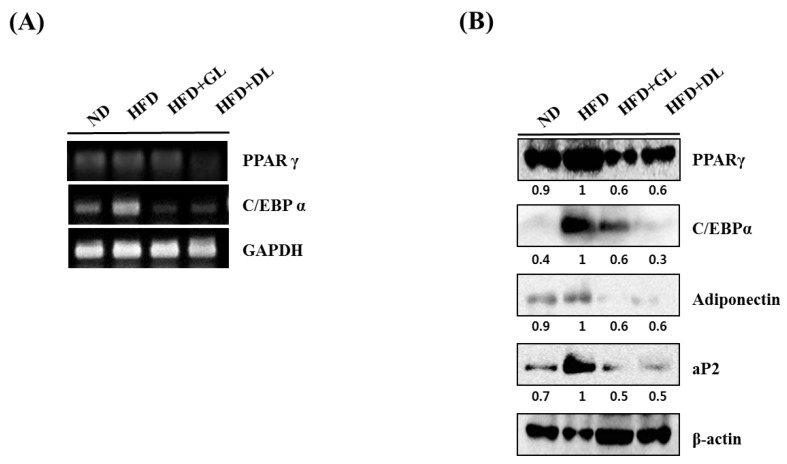
Effects of GL and DL on the mRNA and protein expression of adipogenic genes in adipose tissues. Rats were fed an ND or HFD. Additionally, HFD-fed rats were treated with GL or DL at a dose of 3.3 mg/kg/day. Epididymal WAT was acquired on the final day of the experiments. (**A**) mRNA expression of PPARγ, C/EBPα, and GAPDH in epididymal WAT; (**B**) Protein expression of the indicated adipogenic genes in epididymal WAT. Photographs are representative pictures from three independent experiments.

To investigate the mechanisms underlying the anti-obesity effects of GL and DL, we measured the mRNA and protein expression of adipogenesis-related genes such as the key adipogenic regulators C/EBPα and PPARγ. The mRNA and protein expression levels of C/EBPα and PPARγ were significantly lower in the epididymal adipose tissues of HFD-GL and HFD-DL rats than in the HFD-fed rats (Figure 3A,B). In addition, protein expression of the downstream target genes of C/EBPα and PPARγ, including aP2 and adiponectin, decreased in both HFD-GL and HFD-DL rats (Figure 3B).

### 3.4. Effects of GL and DL on Proliferation of 3T3-L1 Adipocytes

To investigate whether the in vivo anti-obesity effects of GL and DL occurred through inhibition of the adipogenesis of adipocytes, we conducted an in vitro experiment using 3T3-L1 adipocytes.

**Figure 4:**
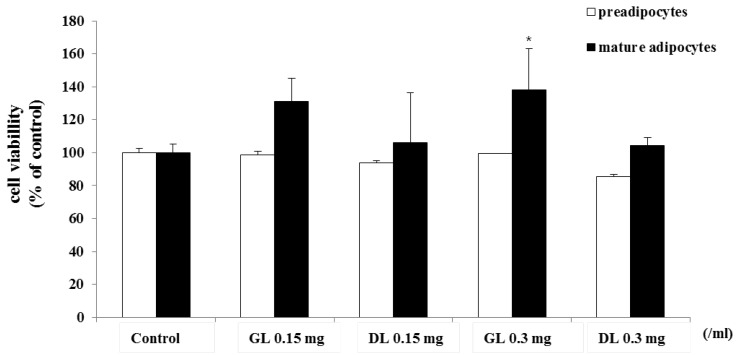
Effects of GL and DL on cell viability of 3T3-L1 preadipocytes and mature adipocytes. 3T3-L1 preadipocytes were treated with GL or DL for 48 h, or left untreated. 3T3-L1 preadipocytes were differentiated into adipocytes and treated with GL or DL for the entire differentiation period or after induction of differentiation. Cell viability was evaluated by using the MTT assay and the absorbance was measured at 495 nm. * *p* < 0.05. Bars represent means ± SD from three independent experiments.

First, we determined the cytotoxicity of GL and DL. The 3T3-L1 adipocytes, as preadipocytes or fully differentiated mature adipocytes, were treated with GL or DL. GL and DL at a concentration of 0.3 g/mL did not affect the viability of 3T3-L1 preadipocytes or fully differentiated mature adipocytes (Figure 4). However, treatment with DL at a concentration of 0.15 mg/mL led to a slight increase in the viability of mature adipocytes.

### 3.5. Effects of GL and DL on Differentiation and Lipid Accumulation of 3T3-L1 Adipocytes

**Figure 5:**
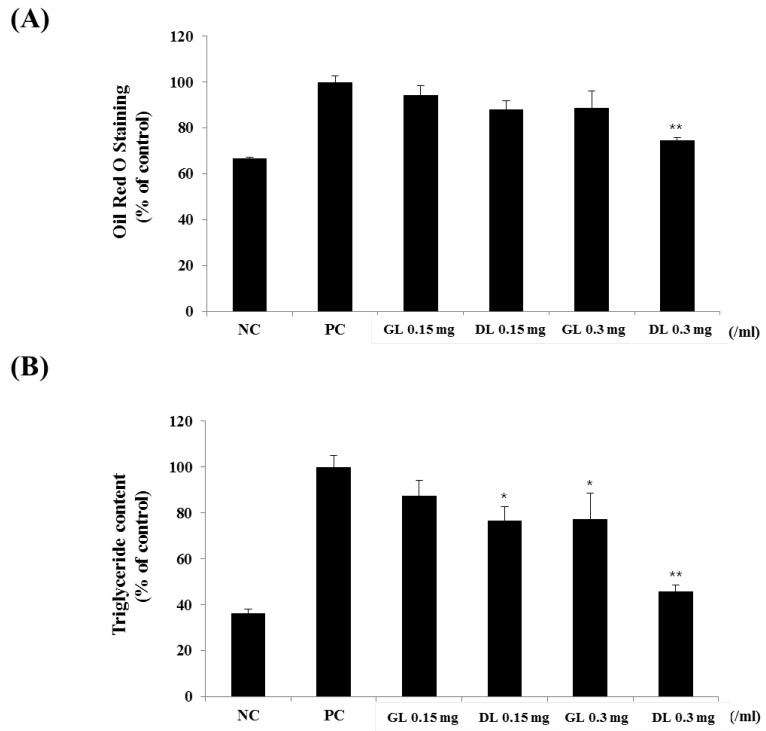
Effects of GL and DL on the differentiation and lipid accumulation of 3T3-L1. 3T3-L1 preadipocytes were treated with GL and DL for eight days, or left untreated (NC). (**A**) The absorbance of cells stained by Oil Red O was measured at 495 nm; and (**B**) intracellular triglyceride concentrations were calculated from measurement of the absorbance at 570 nm. Significant differences from the positive control group (PC), ** *p* < 0.01 or * *p* < 0.05, are indicated. Bars represent means ± SD from three independent experiments.

The 3T3-L1 adipocytes were treated with GL or DL throughout the differentiation period. DL significantly decreased lipid accumulation in a dose-dependent manner (Figure 5A,B). Furthermore, DL treatment at 0.15 and 0.3 mg/mL markedly decreased intracellular TG levels by 15.0% and 30.1%, respectively (Figure 5B). However, GL had little effect on the differentiation of 3T3-L1 adipocytes, which suggested that the in vivo anti-obesity effects of GL were caused by specific factors being released, other organs, or increased energy expenditure.

### 3.6. Effect of GL and DL on the mRNA and Protein Expression of Adipogenic Genes in 3T3-L1 Adipocytes

**Figure 6:**
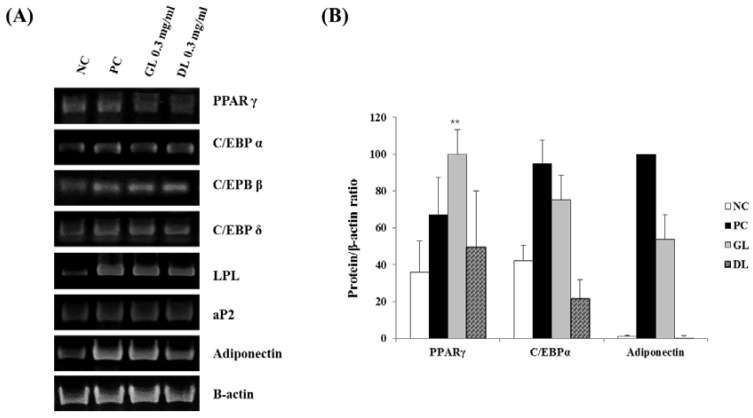
Effects of GL and DL on mRNA and protein expression of adipogenic genes in 3T3-L1 adipocytes. Cells were treated with GL and DL for eight days during differentiation and total RNA and protein content was isolated: (**A**) mRNA expression of the adipogenic genes in 3T3-L1 cells; and (**B**) protein expression of PPARγ, C/EBPα, and adiponectin in 3T3-L1 cells. ** *p* < 0.01. Bars represent means ± SD from three independent experiments.

To investigate the mechanisms underlying the anti-differentiation effects of GL and DL, we measured the mRNA and protein expression levels of adipogenic genes such as the key adipogenic regulators C/EBPs and PPARγ. Treatment with DL significantly decreased the mRNA and protein expression levels of C/EBPα, PPARγ, and adiponectin, and decreased the mRNA expression of fatty acid-binding protein 4 (aP2), lipoprotein lipase (LPL), adiponectin, and C/EBPs (β and δ) (Figure 6A,B). GL only slightly reduced the mRNA and protein expression of adipogenic genes. Remarkably, GL greatly increased the protein expression level of PPARγ compared with that in untreated cells.

## 4. Discussion and Conclusions

A previous review indicated that the root and leaf—stem extracts of ginseng have similar pharmacological effects [10]. Additionally, a number of studies have indicated that the berry extracts of ginseng could be used to treat diabetes in obese mice [15,16,17,18].

In the present study, we found that two kinds of leaf extracts from ginseng exhibited in vivo anti-obesity effects through different mechanisms. GL and DL resulted in a similar reduction of body weight and adipose tissue mass in HFD-induced obese rats. Furthermore, GL and DL were shown to regulate the content of plasma lipoproteins and TGs, and these results indirectly suggest that GL and DL can improve nonalcoholic steatohepatitis caused by obesity. In the case of DL, these effects were thought to be caused by a decrease in the differentiation of adipocytes. The analysis of mRNA and protein expression revealed that DL regulated the adipogenic process in 3T3-L1 adipocytes through modulation of adipogenic gene expression. In contrast, GL showed little effect on the differentiation of adipocytes; however, in cells treated with GL, the protein expression of PPARγ significantly increased compared with that in untreated cells.

PPARγ is mainly expressed in adipose tissues (WAT and BAT) and is a master regulator of adipogenesis as well as a potent metabolic modulator of whole-body lipid metabolism and insulin sensitivity [19,20,21]. Previous studies have reported that mice lacking skeletal muscle PPARγ developed excess adiposity and hepatic insulin resistance [19,21]. Another study indicated that PPARγ hypomorphic mice have mild lipodystrophy due to efficient compensation by other organs such as muscles [22]. Therefore, from a dynamic point of view, we hypothesized that the anti-obesity effects of GL may be caused by improved glucose homeostasis or energy expenditure through greatly increased expression of PPARγ. However, further experiments, such as investigation into glucose homeostatic changes following GL treatment, are required to establish this hypothesis.

Next, we confirmed that treatment with DL at the 0.15 mg/mL concentration increases the viability of mature adipocytes, and this result was confirmed by the MTT assay, based on mitochondrial activity. Beige/brite adipocytes, having thermogenic capacity, show more abundant mitochondria compared to those in white adipocytes [23]. A previous study demonstrated that curcumin exposure induces a brown fat-like phenotype in 3T3-L1 cells [24]. In this regard, although we did not test this, we suggest that the increased viability of mature adipocytes treated with DL was attributable to the change in the phenotype of 3T3-Ll cells. 

In conclusion, our results demonstrated that the leaf extracts of Korean ginseng exhibited anti-obesity effects by selectively reducing adipose tissue mass. DL suppressed the adipogenesis of 3T3-L1 adipocytes through modulation of the expression of central transcription factors. However, the anti-obesity effects of GL were thought to occur through metabolic modulation or increased energy expenditure. However, additional experimentation is needed to further clarify this issue. To the best of our knowledge, these findings are the first evidence for the anti-obesity effects of the leaf extracts of Korean ginseng in vivo, and we propose that leaf extracts of Korean ginseng can be used a potential therapeutic agent or dietary supplement against obesity.

## Supplementary Materials

The following are available online at www.mdpi.com/2072-6643/9/9/999/s1, Table S1: The composition of experimental diets; Table S2: Sequences and accession numbers for primers used in RT-PCR; Table S3: Plasma biochemical values in male rats; Table S4: Effects of green leaf (GL) and dried leaf (DL) on the weights of liver, kidney, heart, spleen, pancreas, and muscle tissues in male rats.
