# Supplementary material for: Panax ginseng Leaf Extracts Exert Anti-Obesity Effects in High-Fat Diet-Induced Obese Rats
Source: Nutrients. 2017 Sep 10;9(9):999. doi: 10.3390/nu9090999 (PMC5622759; doi:10.3390/nu9090999)
Supplement: Supplementary file 1 [file nutrients-09-00999-s001.docx]

Supplementary Materials: Panax ginseng Leaf Extracts Exert Anti-Obesity Effects in High-Fat Diet-Induced Obese Rats

**Seul-Gi Lee, Yoon-Jeong Lee, Myeong-Hwan Jang, Tae-Ryong Kwon and Ju-Ock Nam**

**Table S1.** The composition of experimental diets.

Corn starch

Casein

Sucrose

Cellulose

Mineral mix

Vitamin mix

Choline

ND

HFD

400

200

200

200

140

200

50

50

35

35

10

10

1.5

2

Ingredients

(g/kg of diet)

Corn oil

60

210

Lard

40

150

D,L-methionine

3.5

3

**Table S2.** Sequences and accession numbers for primers used in RT-PCR.

Gene name

PPAR γ (M)

β-actin (M)

C/EBPβ (M)

C/EBPδ (M)

aP2 (M)

Adiponectin (M)

LPL (M)

AACACCGAGATTTCCTTCAA

Accession no.

Forward primer

Reverse primer

C/EBPα (M)

TCACGCCTTTCATAACACAT

ACCTACGACCAGTATCAGGAAAAG

ACTAAGCTGAAAGTGTGTCGACTG

TTACAACAGGCCAGGTTTCC

GGCTGGCGACATACAGATCA

GACTACGCAACACACGTGTAACT

CAAAACCAAAAACATCAACAACCC

GATCTGCACGGCCTGTTGTA

CTCCACTGCCCACCTGTC

TCCTCTGACATTTGCAGGTCTATC

TCACGCCTTTCATAACACAT

TTTTCAAGGGTGCCAGTTTC

AATCCTTGGCCCTCTGAGAT

GACAACGGCTCCGGCATGTGCAAAG

TTCACGGTTGGCCTTAGGGTTCAG

NM_024406

NM_009605

NM_001287523

NM_009883

NM_007679

NM_008509

EF095208

AB644275

M, Mouse; R, Rat

C/EBPα (R)

NM_001287579

GCCAAGAAGTCGGTGGATAA

CCTTGACCAAGGAGCTCTCA

PPAR γ (R)

NM_013124

CTTGGCCATATTTATAGCTGTCATTATT

TGTCCTCGATGGGCTTCAC

GAPDH (R)

TCTGACATGCCGCCTGGAGAA

TGGAGGCCATGTAGGCCATGA

NM_017008

**Table S3.** Plasma biochemical values in male rats.

ALB

Tbil

BUN

CRE

(g/dL)

(mg/dl)

(mg/dl)

(mg/dl)

ND

HFD

HFD+GL

HFD+DL

2.2±0.06

2.3±0.18

2.1±0.28

2.2±0.29

0.2±0.02

0.2±0.02

0.2±0.02

0.2±0.02

22.9±4.77

19.2±5.59

20.5±4.91

28.0±3.80

0.3±0.03

0.3±0.07

0.3±0.06

0.3±0.01

ABL: abetalipoproteinemia, TbiL: bilirubin, BUN: blood-urea nitrogen, CRE: creatinine

**Table S4.** Effects of green leaf (GL) and dried leaf (DL) on the weights of liver, kidney, heart, spleen, pancreas, and muscle tissues in male rats.

Liver weight

(g)

ND

HFD

HFD+GL

HFD+DL

4.9±0.51

7.3±1.40

4.7±0.67

5.2±0.49

1.7±0.07

1.9±0.12

1.7±0.11

1.8±0.18

0.7±0.05

0.7±0.13

0.8±0.07

0.6±0.03

0.3±0.06

0.3±0.04

0.4±0.03

0.3±0.08

Kidney weight

(g)

Heart weight

(g)

Spleen weight

(g)

Muscle weight

Gastrocnemius

(g)

Soleus

(g)

0.1±0.02

2.0±0.13

Pancreas

(g)

0.6±0.10

0.2±0.01

2.0±0.28

0.4±0.17

0.1±0.01

1.9±0.32

0.8±0.16

0.2±0.01

1.8±0.21

1.0±0.10

*

*

Significant differences from the high-fat diet group (HFD), P < 0.01(**) and P < 0.05(*). Data represent means ± SD (n = 7).
